# Fatigue in Cancer and Neuroinflammatory and Autoimmune Disease: CNS Arousal Matters

**DOI:** 10.3390/brainsci10090569

**Published:** 2020-08-19

**Authors:** Christine Ulke, Galina Surova, Christian Sander, Christoph Engel, Kerstin Wirkner, Philippe Jawinski, Tilman Hensch, Ulrich Hegerl

**Affiliations:** 1 Department of Psychiatry and Psychotherapy, Leipzig University Medical Center, Semmelweisstrasse 10, D-04103 Leipzig, Germany; surova_galina@rambler.ru (G.S.); Christian.Sander@medizin.uni-leipzig.de (C.S.); Tilman.Hensch@medizin.uni-leipzig.de (T.H.); 2 LIFE—Leipzig Research Center for Civilization Diseases, University of Leipzig, D-04103 Leipzig, Germany; christoph.engel@imise.uni-leipzig.de (C.E.); kwirkner@life.uni-leipzig.de (K.W.); philippe.jawinski@hu-berlin.de (P.J.); 3 Department of Psychology, Humboldt-Universität zu Berlin, 10099 Berlin, Germany; 4 Department of Psychology, IUBH International University, 99084 Erfurt, Germany; 5 Department of Psychiatry, Psychosomatic Medicine and Psychotherapy, Goethe-Universität Frankfurt am Main, 60323 Frankfurt, Germany; Ulrich.Hegerl@kgu.de

**Keywords:** depression, fatigue, electroencephalography, hyperarousal, neurophysiology

## Abstract

The term fatigue is not only used to describe a sleepy state with a lack of drive, as observed in patients with chronic physical illnesses, but also a state with an inhibition of drive and central nervous system (CNS) hyperarousal, as frequently observed in patients with major depression. An electroencephalogram (EEG)-based algorithm has been developed to objectively assess CNS arousal and to disentangle these pathophysiologically heterogeneous forms of fatigue. The aim of this study was to test the hypothesis that fatigued patients with CNS hyperarousal score higher on depressive symptoms than those without this neurophysiological pattern. Methods: Subjects with fatigue (Multidimensional Fatigue Inventory sum-score > 40) in the context of cancer, neuroinflammatory, or autoimmune diseases were drawn from the 60+ cohort of the Leipzig Research Center for Civilization Diseases. CNS arousal was assessed by automatic EEG-vigilance stage classification using the Vigilance Algorithm Leipzig (VIGALL 2.1) based on 20 min EEG recordings at rest with eyes closed. Depression was assessed by the Inventory of Depressive Symptomatology (IDS-SR). Results: Sixty participants (33 female; median age: 67.5 years) were included in the analysis. As hypothesized, fatigued patients with CNS hyperarousal had higher IDS-SR scores than those without hyperarousal (F_1,58_ = 18.34; *p* < 0.0001, η^2^ = 0.240). Conclusion: hyperaroused fatigue in patients with chronic physical illness may be a sign of comorbid depression.

## 1. Introduction 

Fatigue is a widespread symptom among patients with cancer, neuroinflammatory or autoimmune disease. For example, severe chronic fatigue is reported in 41–57% of patients with rheumatoid arthritis [1], and in 67% of patients with Sjogren’s syndrome [2]. Among cancer patients, the prevalence rate ranges from 59% to 100%, depending on the clinical status of the cancer [3]. In patients with Parkinson’s disease, prevalence rates between 37% and 57% [4] have been reported. Moreover, more than 80% of multiple sclerosis patients suffer from intractable chronic fatigue within the first year of disease onset [5]. 

However, fatigue is also a symptom and prodromal symptom in major depression (MD; [6]), which is a frequent comorbid diagnosis in patients with the somatic disorders mentioned above, with estimated prevalence rates of between 17% and 40% [7,8,9,10]. Evidence has been provided that fatigue in the context of inflammatory and immunological processes should not be confused with fatigue in MD, because they show profound neurophysiological differences—especially concerning central nervous system (CNS) arousal [11,12]. A differentiation of these distinct forms of fatigue along the dimension of CNS arousal may have implications for treatment [11,12,13]. Transdiagnostically, the dimension of arousal gained relevance within a new research framework for studying mental illnesses—the Research Domain Criteria (RDoC) Project [14]—wherein arousal and its regulation constitute one of the five fundamental dimensions. The term *brain arousal* refers to global brain states which, on a behavioral level, vary along a continuum between wakefulness and sleep. It can be assessed using a resting state electroencephalogram (EEG; [15,16]) by automatic EEG-vigilance stage classification with the Vigilance Algorithm Leipzig (VIGALL; [17]; for the validation of VIGALL, see [18,19,20,21,22]). Resting state eyes-closed EEG has been extensively used to study CNS arousal, providing data largely free of eye-movement artifacts and not confounded by a subject’s ability (and motivation) to maintain wakefulness, as required by eyes-open or eyes fixating on a cross paradigms. The classification with VIGALL takes into account frequency patterns and the cortical distribution of EEG activity by utilizing low resolution electromagnetic tomography (LORETA; [23]). Using this tool, inter-individual differences in the regulation of brain arousal during a 20 min resting state EEG can be discerned. While most individuals show progressive declines to lower EEG-vigilance stages (i.e., drowsy or sleepy) during the resting state EEG, others remain in a state of high arousal (i.e., alert or hypervigilant) throughout the recording. Such hyperarousal is a frequent finding in medicated and unmedicated patients with MD [24,25,26,27], and it has been shown to correlate with symptom severity in MD patients [27]. In addition, at the gene level, a genome-wide association study (GWAS) revealed significant evidence of an association of brain arousal with TMEM159, with other GWAS analyses and gene expression data suggesting a role of TMEM159 in MD [28]. Other indicators of hyperarousal in MD, such as prolonged sleep onset latencies [29,30], increased heart rate and skin conductance levels [31], or hyperactivity of the hypothalamic-pituitary-adrenal (HPA)-axis [32], are in line with the view that arousal may play a significant pathophysiological role in MD.

In contrast to the hyperarousal frequently observed in depression, evidence has been provided that fatigue in the context of somatic disorders may be associated with hypoarousal. This is supported by previous findings of a fast decline to low arousal levels during a 15 min EEG at rest in fatigued cancer patients [33]. It is also corroborated by the inflammatory response model of fatigue [34], suggesting an upregulation of proinflammatory and sleep-promoting cytokines (i.e., Interleukin-1 beta (IL-1) and tumor necrosis factor-α (TNF-α)) in fatigued patients with physical illness. Indeed, the association of IL-1 and TNF-α with fatigue has been demonstrated in several medical conditions [35,36]. Additionally, associations between TNF-α and sleepiness have been reported [25,37].

In view of these findings, we have proposed to define two subtypes of fatigue based on their assumed underlying neurobiological mechanisms concerning arousal regulation [11,12]—hyperaroused fatigue with reduced sleep propensity, inhibition of drive and exhaustion (typical of depression), and hypoaroused fatigue with increased sleepiness, a lack of drive, and sickness behaviour, typical in context of inflammatory and immunological processes [11].

Up to now, there has been little research on the association between depressive symptoms and CNS arousal in patients with fatigue and physical illness. The question is justified because of the high prevalence rates of comorbid depression. There is first evidence that hyperaroused fatigue in these conditions may be accompanied by CNS hyperarousal. For example, although fatigue in multiple sclerosis has been reported to co-occur with excessive daytime sleepiness [38,39] and short sleep onset latencies in the multiple sleep latency test (MSLT; [39]), HPA-axis upregulation in multiple sclerosis patients with fatigue was also reported [40]. Furthermore, increased HPA-axis activity was found to be associated with hyperstable arousal regulation in medicated patients with multiple sclerosis [41]. Therefore, based on the accumulated evidence outlined above, we expect that CNS hyperarousal would be indicative of depression.

The aim of this study was to test the hypothesis that fatigued patients with CNS hyperarousal have a higher depression score than those without this neurophysiological pattern. Participants with cancer or neuroinflammatory or autoimmune disease—complaining about fatigue—were selected from a population-based study, the Leipzig Research Center for Civilization Diseases (LIFE)-Adult-Study [42]. All had undergone a comprehensive medical assessment, including a 20 min eyes-closed resting EEG. The Vigilance Algorithm Leipzig (VIGALL 2.1, licensed under GPL3) was used to score the EEG-vigilance stages (indicating arousal states) for each 1 s epoch of the resting EEG. The individual arousal regulation type was determined based on the time course of the arousal stages. Individuals with and without CNS hyperarousal were compared regarding their depression score and, within sensitivity analyses, concerning their depressive symptom profiles.

## 2. Materials and Methods

### 2.1. Sample

The participants were drawn from the 60+ LIFE cohort of the LIFE-Adult-Study, a population-based cohort study in Leipzig, Germany [42]. All participants had given written informed consent, and all the procedures were conducted according to the Declaration of Helsinki and were approved by the Ethics Committee of the University of Leipzig (263-2009-14122009). We selected study participants (age range: 60–70) with cancer or neuroinflammatory or autoimmune disease (to be present within the last 12 months and/or treated at time of assessment), with a sum-score of >40 in the Multidimensional Fatigue Inventory (MFI-20; [43]), who had undergone a 20 min resting-state EEG (*n* = 72). We excluded participants with substance dependence; participants with psychotic, affective, or anxiety disorders (assessed with the Structured Clinical Interview for DSM-IV axis I disorders; [44]); and participants on antidepressant medication, benzodiazepines, and z-hypnotics. We also excluded participants with pathological EEG activity, low-voltage alpha or alpha-variant rhythms, or with substantial artifacts in the EEG. The final sample consisted of 60 participants (33 female; median age: 67.5).

### 2.2. Questionnaires

Fatigue was assessed with the MFI-20, a 20-item self-rating questionnaire consisting of five fatigue dimensions: general fatigue, physical fatigue, mental fatigue, reduced activity, and reduced motivation [43]. Each dimension contains four items ranging from 1 to 5, with higher scores indicating a higher degree of fatigue. A sum-score over 40 was used as cut-off-point, analogous to the study by Olbrich et al. [33], in which the presence of fatigue in cancer patients with a MFI cut-off-point above 40 was validated by a senior physician.

The sum-score of the self-rating version of the Inventory of Depressive Symptomatology (IDS-SR; [45]), a 30-item questionnaire, was used to assess the severity of depression (no depressive symptomatology: IDS-SR < 14; mild depressive symptom severity: 14 ≤ IDS-SR < 26; moderate depressive symptom severity: 26 ≤ IDS-SR < 39; severe depressive symptom severity: IDS-SR ≥ 39).

Sleep quality and sleep duration were assessed using the German version of the Pittsburgh Sleep Quality Index (PSQI; [46]), which assesses subjectively rated sleep. The PSQI is a 19-item questionnaire designed to measure sleep quality and disturbance over the past month in clinical populations. The sleep component scores are summed to yield a total score ranging from 0 to 21, with a higher total score indicating worse sleep quality [46]. Trait daytime sleepiness was assessed with the Epworth Sleepiness Scale (ESS; [47]), an 8-item questionnaire, with scores over 10 indicating mild excessive daytime sleepiness [48].

### 2.3. EEG Recording and Preprocessing

EEGs were recorded and processed according to a standard operating procedure [17]. During the recording, the participants lay on a semi-reclined lounge chair in a sound- and light attenuated booth. Before the recording, the participants were asked to complete a simple arithmetic task (counting backwards by sixes starting at 100) to ensure similar arousal levels between subjects. Thereafter, they were instructed to close their eyes and to relax during the 20 min resting condition. Electroencephalographic and electro-oculographic activities were recorded with a sampling rate of 1000 Hz from 31 electrode sites, following an extended 10–20 system using a QuickAmp-40 amplifier (Brain Products GmbH, Gilching, Germany). Impedances were kept below 10 kΩ. EEG offline processing was conducted in BrainVision Analyser 2.1 (Brain Products GmbH, Gilching, Germany). After filtering (0.5 Hz high-pass, 70 Hz low-pass, with a 48 dB/Oct slope and 50 Hz notch filter), the data were segmented into 1 s epochs and down-sampled to 500 Hz for further preprocessing (and again to 100 Hz immediately before VIGALL application). Graphoelements indicating sleep onset (sleep spindles, K-complexes) were marked by experienced raters according to set criteria, as these markers are used by VIGALL to classify C-stages. Using independent component analysis (ICA; [49]), components corresponding to muscle, sweating, cardiac, and eye movement artifacts were removed (in most cases, 2 eye and 1 cardiac component and up to 5 components containing muscle or other artifacts, depending on which components were found). Afterwards, segments with remaining artifacts (approximately 2% of all segments) were marked (and not classified by VIGALL).

### 2.4. EEG-Vigilance Staging and Parameterization

To assess CNS arousal, we applied VIGALL 2.1 to the 20 min EEG to classify each of the 1200 1 s segments into one-out-of-seven EEG-vigilance stages, ranging from active wakefulness (Stage 0) to sleep onset (Stage C), including Stage 0 (low-voltage EEG without slow horizontal eye movements), Stage A (dominant alpha rhythm; cortical areas: A1 (occipital); A2 (central); A3 (frontal)), Stage B1 (low-amplitude non-alpha EEG with slow horizontal eye movements), Stage B2/3 (high delta and theta power), and Stage C (occurrence of sleep spindles, K-complexes, sleep onset). Subsequently, using the scoring criteria presented in Table 1, we calculated an Arousal Stability Score [30] for each participant, to assess the degree of arousal decline during the 20 min resting EEG. We divided the patients into two groups: patients with hyperaroused fatigue (Arousal Stability Score ≥ 13) and patients with non-hyperaroused fatigue (Arousal Stability Score ≤ 12). The rationale for this cut-off was based on previous findings in depressed patients [24,25], who remained in high EEG-vigilance stages during an EEG at quiet rest (indicating hyperarousal). An Arousal Stability Score of 13 or 14 indicates that more than 2/3 of all the segments in each of the twenty 1 min epochs were classified as at least Stage A or higher (Table 1).

### 2.5. Statistical Analyses

Statistical analyses were conducted in SPSS Statistics 24 (IBM corp., Armonk, NY, USA). To identify the relevant covariates for the between-group comparison of the depression score, we analyzed the differences in demographic and clinical variables between the arousal groups using parametric (*t*-test) or nonparametric tests (Chi^2^, Mann–Whitney U) depending on data level and their distribution. The term *gender* was defined as male or female sex. For the main analysis, an analysis of variance (ANOVA) was utilized to calculate the between-group differences in the IDS-SR sum-score. The significance level was set at *p* = 0.05 (one-tailed). For the exploratory sensitivity analyses, we employed two-tailed tests.

## 3. Results

### 3.1. Descriptive Analyses

The characteristics of the total sample and of the arousal subgroups are described in Table 2. Of the 60 study participants, 31.7% (*n* = 19) had an Arousal Stability Score of 13 or higher, indicating that they remained in high arousal states during the 20 min EEG. Those 19 participants were assigned to the hyperaroused fatigue subgroup; all the others (*n* = 41) were assigned to the non-hyperaroused fatigue subgroup (cf. Table 2). Forty percent of the participants (*n* = 24) did not report any depressive symptoms, 53.3% (*n* = 32) had mild ones, and 6.7% (*n* = 4) had moderate to severe depressive symptom severity. The hyperaroused subgroup did not differ from the non-hyperaroused subgroup in regard to age, type of physical illness, daytime sleepiness, and severity of fatigue (*p* < 0.390). There was a statistical trend for significant differences concerning gender (χ^2^ = 3.92; df = 1; *p* = 0.057), wherein female participants tended to have higher arousal levels than male participants. There were no group differences concerning the time of day of the EEG recording, coffee consumption prior the EEG recording, and daytime sleepiness (cf. Table 3). The self-rated sleep quality, as assessed by the PSQI total score, differed between groups (Z = −2.78; *p* = 0.005), indicating worse sleep quality in the hyperaroused as compared to the non-hyperaroused subgroup. However, the frequency distribution of PSQI phenotypes (cf. Table 3) did not differ between the groups (χ^2^ = 3.680; *p* = 0.159).

### 3.2. Main Analysis: Between-Group Comparisons of the Depression Score

**Figure 1:**
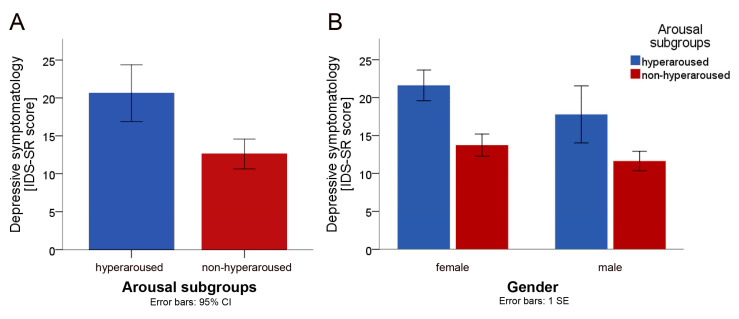
Between-group comparisons (hyperaroused (*n* = 19) vs. non-hyperaroused (*n* = 41)). (**A**) Between-group comparison of the mean sum-score of the Inventory of Depressive Symptomatology (IDS-SR). (**B**) Between-group comparison of the IDS-SR means, stratified by gender.

The hyperaroused subgroup (IDS-SR sum-score: 20.6 ± 7.3, range 10–41) scored higher on depression than the non-hyperaroused subgroup (IDS-SR sum-score: 12.6 ± 6.2, range 1–24). We conducted a one-way ANOVA to assess the level of depressive symptomatology across the subgroups (hyperaroused vs non-hyperaroused). Levene’s test of significance indicated that the assumption of homogeneity of variance was met (F_(1, 58)_ = 0.559; *p* = 0.458). The main group effect was significant (F_(1, 58)_ = 18.340; *p* < 0.0001; η^2^_p_ = 0.240), indicating that patients with hyperaroused fatigue scored significantly higher on the IDS-SR than patients with non-hyperaroused fatigue (cf. Figure 1A). To control for gender, a two-way ANOVA was conducted with gender as additional factor (respective means in either group are presented in Figure 1B). Levene’s test for the equality of variances was met (F_(3, 56)_ = 0.089; *p* = 0.966). The main group effect was still significant (F_(1, 56)_ = 11.80; *p* = 0.001; η^2^_p_ = 0.174) when gender was included as a factor in the model, whereas the effect of gender was not significant (F_(1, 56)_ = 2.150; *p* = 0.152; η^2^_p_ = 0.036), indicating that gender had no significant effect on the level of depressive symptomatology. Furthermore, there was no significant interaction between group and gender (F_(1, 56)_ = 0.181; *p* = 0.672; η^2^_p_ = 0.003).

Post-hoc receiver operating characteristic (ROC) analyses, examining arousal stability (at the cut-off score of 13) as a diagnostic screening test for moderate to severe depression, revealed a 0.87 area under the curve (AUC; *p* = 0.014; 95% CI 0.765–0.976), indicating a sensitivity of 100%, a specificity of 73%, and a Youden Index of 0.7 [50].

### 3.3. Exploratory Sensitivity Analyses: Between-Group Comparisons of IDS-SR Items

To assess whether the arousal groups differ concerning typical depressive symptoms, we conducted Mann–Whitney U (MWU) tests. MWU tests revealed significant differences between the hyperaroused and the non-hyperaroused subgroups in items initial insomnia (Z = −3.803, *p* < 0.001), early morning awakening (Z = −3.368, *p* < 0.0001), feeling sad (Z = −2.517, *p* = 0.012), feeling anxious or tense (Z = −2.874, *p* = 0.004), increased appetite (Z = −2.202, *p* = 0.028), concentration/decision-making (Z = −3.070; *p* = 0.002), self-criticism/blame (Z = −2.361, *p* = 0.018), energy level (Z = −2.940, *p* = 0.003), psychomotor retardation (Z = −3.033, *p* = 0.002), and agitation (Z = −3.046, *p* = 0.002). Relative to individuals in the non-hyperaroused group, individuals in the hyperaroused group took a longer time to fall asleep and woke up too early; felt more often sad, anxious, or tense; felt more often slowed down or restless; had more frequently a negative view of themselves; experienced more often concentration and decision-making problems; had an increased appetite and a lower energy level.

## 4. Discussion

The aim of the current study was to test the hypothesis that fatigued patients with chronic physical illnesses, and CNS hyperarousal during a 20 min EEG at rest, have a higher depression score than those without this neurophysiological pattern. The hypothesis was confirmed; individuals with hyperaroused fatigue had significantly higher depression scores than those with non-hyperaroused fatigue; an eta-squared value of over 0.2 indicated a large effect size. The between-group differences in depression scores remained significant when gender was included as a factor in the model. In sensitivity analyses, individuals in the hyperaroused group achieved higher scores in a series of depressive symptoms, such as depressed mood, insomnia, and concentration/decision-making problems, as compared to individuals in the non-hyperaroused group.

Our results are in line with a previous study examining arousal regulation in multiple sclerosis patients with fatigue, wherein evidence for distinct pathophysiological correlates of fatigue was provided [41]. In this study, the hyperaroused fatigue subtype, as assessed by EEG-vigilance using VIGALL, corresponded to the highest HPA-axis activity, while the hypoaroused fatigue subtype revealed the lowest HPA-axis activity [41]. These findings, as the results of the current study, corroborate the validity of the distinction of fatigue subtypes along the axis of brain arousal and of CNS hyperarousal as a state marker of depression [11,12,24].

Group differences in the IDS-SR total score between patients with and without hyperarousal, as revealed by exploratory item comparisons, emerged not merely due to confounding symptoms of the underlying disease, such as pain, weight changes, or negative view of the future. Instead, individuals in the hyperaroused group achieved higher scores in a series of typical symptoms of depression, including depressed mood, concentration/decision-making difficulties, and sleep and psychomotor changes. An inconsistency was the reported increased appetite in the hyperaroused group, which is more often associated with the atypical depressive subtype [51]. A possible explanation consists in the fact that our sample was composed of individuals with chronic physical illnesses, which may be accompanied by changes in appetite by themselves. Although subjects with current major depression, as assessed by a structured clinical interview, were excluded, 7% of the sample complained about moderate to severe depressive symptoms, possibly revealing discrepancies between the self-rating and physician-rating scales. Further, 53% of the sample reported mild depressive symptoms. To date, an association between hyperarousal and depressive symptoms has been frequently demonstrated in the context of major depression [24,25,26]; our results suggest that this association could exist within subthreshold depressive symptomatology.

The sleep quality, assessed with the PSQI, differed significantly between the arousal groups—hyperaroused individuals reported worse sleep quality. In contrast, daytime sleepiness, assessed with ESS, was comparable. In healthy individuals, one would expect that poor sleep at night would be associated with increased daytime sleepiness and CNS hypoarousal. In patients with major depression, however, CNS hyperarousal is considered an important pathogenetic mechanism leading to both sleep problems and increased brain arousal during the day [30,52]. This could explain the co-occurrence of poor sleep quality and high daytime arousal in the current study.

Three limitations should be mentioned: (i) the presence of chronic physical diseases was assessed via self-reporting without validation by a medical specialist; (ii) the applied questionnaires (physical disease diagnoses) were designed specifically for the LIFE study and have not been externally validated; (iii) the advanced age of the sample (median age: 67.5 years) limits the generalizability of the study.

Despite these limitations, the hypothesis that a subgroup of hyperaroused fatigued patients with somatic disorders might suffer from undetected depression, has been confirmed. Considering the relevancy for treatment decisions, future clinical trials stratifying fatigued patient by patterns of brain arousal for treatment allocation, are warranted.

## Figures and Tables

**Table 1:** Scoring criteria of the Arousal Stability Score. The score quantifies the degree of arousal decline on the basis of subsequently classified 1 s EEG segments with VIGALL 2.1.

Score	Scoring Criteria	EEG Block	Operational Definition
14	More than 2/3 of all segments in each 1 min epoch classified as 0/A1- or 0/A-stages	1–4	Predominant classification of 0 and A1
13		1–4	Predominant classification of 0 and A
12	At least 1/3 of all segments in a 1 min epoch classified as B1-stages	4	Stage B1 emerged in min 16–20
11		3	Stage B1 emerged in min 11–15
10		2	Stage B1 emerged in min 6–10
9		1	Stage B1 emerged in min 1–5
8	At least 1/3 of segments in a 1 min epoch	4	Stage B2/3 emerged in min 16–20
7	classified as B2/3-stages	3	Stage B2/3 emerged in min 11–15
6		2	Stage B2/3 emerged in min 6–10
5		1	Stage B2/3 emerged in min 1–5
4	At least one C-stage classified	4	Stage C emerged in min 16–20
3		3	Stage C emerged in min 11–15
2		2	Stage C emerged in min 6–10
1		1	Stage C emerged in min 1–5

Annotations: EEG block 1 = min 1–5, EEG block 2 = min 6–10, EEG block 3 = min 11–15, EEG block 4 = min 16–20.

**Table 2:** Characteristics of the total sample, and the arousal subgroups.

	All (*n* = 60)	Hyperaroused (*n* = 19)	Non-Hyperaroused (*n* = 41)
**Demographics**			
Age, median years	67.5	68.0	67.0
Sex, f/m (%)	33/27 (55.0/45.0)	14/5 (73.7/26.3)	19/22 (46.3/53.7)
**Cancer**, *n* (%)	30 (50.0)	10 (52.6)	20 (48.8)
Skin cancer	10	4	6
Breast cancer	9	3	6
Prostata cancer	4	0	4
Bladder cancer	2	1	1
Colon cancer	2	1	1
Kidney cancer	1	0	1
Lymphoma	1	1	0
Thyroid cancer	1	0	1
**Neuroinflammatory/autoimmune**, *n* (%)	30 (50.0)	9 (47.4)	21 (51.2)
Rheumatoid arthritis	19	7	12
SLE/Sjogren syndrome	10	1	9
Multiple sclerosis	2	1	1
Parkinson’s disease	2	1	1
**Fatigue**			
MFI, median sum-score	52.0	55.0	51.0

Annotations: MFI = Multiple Fatigue Inventory; SLE = Systemic Lupus Erythematosus.

**Table 3:** Description of arousal-related variables in the total sample, and in the arousal subgroups.

	All (*n* = 60)	Hyperaroused (*n* = 19)	Non-Hyperaroused (*n* = 41)
**Arousal Stability Score**, median	10.0	13.0	9.0
**ESS**, mean score (SD)	7.9 ± 3.4	6.8 ± 3.0	8.3 ± 3.6
**PSQI**, mean score (SD)	6.5 ± 3.5	8.4 ± 3.6	5.6 ± 3.1
Good sleep quality, *n* (%)	26 (47.3)	5 (29.4)	21 (55.3)
Poor sleep quality, *n* (%)	19 (34.6)	7 (41.2)	12 (31.6)
Potentially clinically relevant sleep disorder, *n* (%)	10 (18.2)	5 (29.4)	5 (13.2)
Total time in bed, median hours (range)	8.6 (4.0–12.5)	9.0 (6.5–12.5)	8.5 (4.0–10.0)
**EEG-Related Variables**			
Time of EEG recording, median, hh:mm	9:00	9:00	9:00
Coffee prior to EEG, yes (%)	51 (85.0)	16 (84.2)	35 (85.4)

Annotations: ESS = Epworth Sleepiness Scale; PSQI = Pittsburgh Sleep Quality Index (only *n* = 55 (hyperaroused: *n* = 17; non-hyperaroused: *n* = 38) with available data).
